# Study on the Pharmacological Efficacy and Mechanism of Dual-Target Liposome Complex AD808 Against Alzheimer’s Disease

**DOI:** 10.3390/ph18070977

**Published:** 2025-06-29

**Authors:** Chang Liu, Xiaoqing Wang, Wei Xu, Songli Yu, Yueru Zhang, Qiming Xu, Xiangshi Tan

**Affiliations:** 1 Department of Chemistry, Fudan University, Shanghai 200433, China; 19110220106@fudan.edu.cn (C.L.); 20110220119@fudan.edu.cn (X.W.); 22110220101@fudan.edu.cn (S.Y.); 13110220012@fudan.edu.cn (Q.X.); 2 School of Advanced Materials Engineering, Jiaxing Nanhu University, Jiaxing 314000, China; xuwei@jxnhu.edu.com; 3 Hangzhou Orenstar Biomed Co., Ltd., Hangzhou 314000, China; orenstar@163.com

**Keywords:** innate immune regulation, Alzheimer’s disease, STING/IRF3/PI3K/AKT pathway, microglia, liposome

## Abstract

**Background/Objectives**: To study the efficacy and pharmacological mechanism of the dual-target liposome complex AD808 in the treatment of Alzheimer’s disease. **Methods**: Using APP/PS1 mouse models, the therapeutic efficacy and pharmacological mechanism of AD808 on Alzheimer’s disease were studied through water maze tests, brain tissue staining, immunofluorescence, and ELISA for inflammatory and neurotrophic factors. **Results**: AD808 exhibited significant pharmacodynamic effects in improving behavioral and cognitive abilities (70% reduction in escape latency) and repairing damaged nerve cells (90% reduction in Aβ plaque) in Alzheimer’s disease mice. The efficacy of the liposome complex AD808 was significantly better than that of ST707 or gh625-Zn_7_MT3 alone. AD808 significantly reduced brain inflammation (57.3% and 61.5% reductions in TNF-α and IL-1β, respectively) in AD (Alzheimer’s disease) mouse models and promoted the upregulation of neurotrophic factors and nerve growth factors (142.8% increase in BDNF, 275.9% in GDNF, and 111.3% in NGF-1) in brain homogenates. By activating the PI3K/AKT signaling pathway in brain microglia, AD808 upregulated TREM2 protein expression and removed Aβ amyloid plaques in the brain. Additionally, it promoted the transition of microglia from the pro-inflammatory M1 phenotype to the anti-inflammatory M2 phenotype, regulated the M1/M2 balance, released anti-inflammatory and neurotrophic factors, reduced chronic inflammation, and enhanced neurological repair. Based on these results, the potential pharmacological mechanism of AD808 against Alzheimer’s disease was proposed. **Conclusions**: As a dual-target liposome complex, AD808 has shown promising therapeutic potential in the treatment of Alzheimer’s disease, providing a new strategy for innovative drug development.

## 1. Introduction

With the aging population, the morbidity and mortality of age-related diseases have increased significantly, imposing a heavy economic burden on society. The number of AD (Alzheimer’s disease) patients is increasing annually, severely compromising human health and leading to significant societal challenges [1,2]. According to research data of 2020, there are 15.07 million dementia patients globally, with AD patients accounting for over 60%. Another study reported that annual AD treatment costs reached USD 167.74 billion, with a steady annual increase projected to reach USD 1887.18 billion by 2050 [3,4]. Currently, AD research has identified the following key pathological features: (1) β-amyloid (Aβ) plaques accumulating in extracellular spaces; (2) neurofibrillary tangles (NFTs) formed by hyperphosphorylated tau protein aggregates; (3) significant neuronal loss [5]; (4) chronic neuroinflammation. Although AD’s pathological features are well defined, its exact pathogenesis remains unclear, and no definitive treatment exists.

Research into AD’s pathological mechanisms has advanced significantly in recent years. A key pathological feature is senile plaques, primarily composed of amyloid-β (Aβ). Aβ aggregates into neurotoxic oligomers in the extracellular space [6]. Aβ also triggers neuroinflammation, disrupts the blood–brain barrier (BBB), and exacerbates AD symptoms [7]. Tau protein hyperphosphorylation is another hallmark of AD pathology. Tau is a microtubule-associated protein essential for axonal stability. Tau contains multiple phosphorylation sites. In healthy individuals, phosphorylation and dephosphorylation remain balanced, whereas in AD brains, hyperphosphorylation predominates. Hyperphosphorylated tau loses axonal stabilization capacity, forming NFTs [8]. Toxic β-amyloid and tau activate microglia (innate immune cells) to clear toxic proteins and cellular debris. When microglia become overwhelmed, chronic neuroinflammation ensues, perpetuating pro-inflammatory cytokine release (e.g., IL-1β, IL-6, TNF-α). This inflammatory response exacerbates neurotoxicity and accelerates Aβ deposition by suppressing insulin-degrading enzyme activity [9].

It was confirmed that transition metal ions (Cu^2+^, Fe^2+^, Zn^2+^) in AD patients’ senile plaques reach concentrations as high as 1 mM, termed a metal ion pool, significantly exceeding physiological levels in the brain microenvironment. These metal ions promote Aβ deposition and tau hyperphosphorylation and potentiate Aβ-mediated neurotoxicity. Studies reveal a significant zinc deficiency in elderly and AD brains, with evidence suggesting that this deficiency promotes senile plaque formation [10]. Copper-containing metalloenzymes in the nervous system participate in redox processes. In AD brain tissue, amyloid deposition coincides with copper ion accumulation, creating localized copper deficiency in the microenvironment. Furthermore, both APP and Aβ bind copper, exerting substantial neurotoxicity through ROS generation while inducing neuroinflammation [11]. Thus, disruption of metal ion homeostasis in the brain microenvironment represents a key contributor to neuronal apoptosis. Metallothionein-3 (MT3) effectively regulates nervous system metal homeostasis, yet studies demonstrate a 30% reduction in MT3 expression in AD brains [12]. MT3 administration in animal models significantly alleviated AD symptoms, confirming this pathway’s therapeutic relevance [13]. Beyond these mechanisms, AD pathogenesis hypotheses include energy metabolism dysregulation, mitochondrial dysfunction, and oxidative stress. These factors may contribute to AD development. Therefore, AD’s pathogenesis remains highly complex and not fully elucidated.

As AD’s etiology and mechanisms remain unclear, effective treatment strategies are lacking, creating significant drug development challenges. Historically, AD drugs have primarily provided symptomatic rather than etiological treatment, offering palliative, not curative, effects. To date, no treatment can completely cure Alzheimer’s disease. Current AD medications (e.g., acetylcholinesterase inhibitors, memantine, donepezil, rivastigmine, galantamine, huperzine A) only alleviate symptoms without effectively delaying disease progression. Lecanem Ab received approval in the US, Japan, and China for AD-related cognitive impairment and mild dementia, marking the first FDA fully approved AD disease-modifying therapy in two decades. However, its efficacy in early AD requires further evaluation, while demonstrating significant side effects and limited neurorestorative capacity. Clinically, drugs effectively eliminating chronic inflammation or restoring tissue function remain unavailable.

Therefore, developing more effective anti-AD therapeutics represents an urgent unmet medical need. Neuroinflammation constitutes a hallmark pathological feature of Alzheimer’s disease, characterized by chronic activation of microglia and sustained release of pro-inflammatory cytokines. Amyloid plaque-induced microglial overactivation exacerbates tissue damage, compromises blood–brain barrier integrity, and elevates oxidative stress, ultimately triggering widespread neuronal apoptosis. Conversely, properly regulated microglial activation plays essential roles in tissue repair and neural remodeling. Therefore, maintaining the homeostatic balance between pro-inflammatory M1 and anti-inflammatory M2 microglial phenotypes is critical. Therapeutic strategies that modulate microglial polarization to promote anti-inflammatory factor secretion and neurotrophic factor release may offer promising approaches for AD treatment [14]. In AD mouse models, TREM2 deficiency exacerbates Aβ accumulation and neuronal degeneration [15]. TREM2 overexpression in AD models reduces Aβ deposition, ameliorates neurite dystrophy, improves behavioral deficits, and attenuates neuroinflammation [15,16,17,18]. Xu et al. demonstrated that cyclic GMP-AMP (cGAMP), an innate immunomodulator, ameliorates cognitive deficits and reduces Aβ plaque burden in AD rats [19]. The cGAMP-STING-IRF3 pathway upregulates the triggering receptor expressed on myeloid cells 2 (TREM2), thereby reducing Aβ deposition [19]. TREM2 promotes microglial polarization from the M1 to the M2 phenotype, attenuating AD-associated neuroinflammation [20]. Furthermore, disrupted metal homeostasis represents another key pathological feature of AD. Xu et al. reported that astrocyte-derived Zn_7_MT3, a metalloregulatory protein, restores metal homeostasis and mitigates oxidative stress in AD brains [13].

The goal of this study is focused on the pharmacological efficacy and mechanism of AD808 against Alzheimer’s disease. AD808 is a dual-target co-loaded liposome complex containing two active ingredients, including an immunomodulatory agent ST707 encapsulated within liposomes, and a recombinant metalloprotein gh625-Zn_7_MT3 connected to the surface of liposomes through covalent bonds. The complex AD808 can release the innate immune modulator ST707 in a sustained manner, synergistically regulate the metal ion homeostasis mediated by Zn_7_MT3, and increase the neurotransmitter zinc ion level, thereby achieving synergistic anti-AD effects through a dual-target mechanism. To verify this effect, these two active ingredients were used as positive controls when studying the pharmacological effects of AD808.

## 2. Results

### 2.1. Characterization of AD808 Liposomal Complex

**Figure 1:**
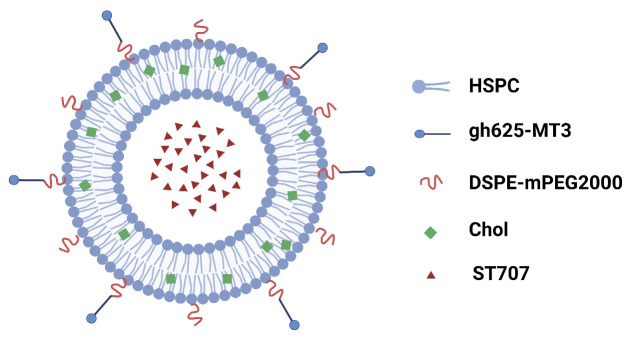
Schematic structure of AD808, showing ST707 encapsulated in the liposomal core and gh625-Zn_7_MT3 conjugated to the bilayer surface. Figure created in BioRender (Premium). (https://app.biorender.com/illustrations/682a7bc4b6e59bb1fe74e7d1?slideId=532d4c21-001f-43c3-a2d3-6512ab3b883e, accessed on 30 March 2025).

The structure of the dual-target liposome AD808 is shown in Figure 1. ST707 is encapsulated within the aqueous core, while the gh625-Zn_7_MT3 protein is conjugated to the surface of the phospholipid bilayer.

**Figure 2:**
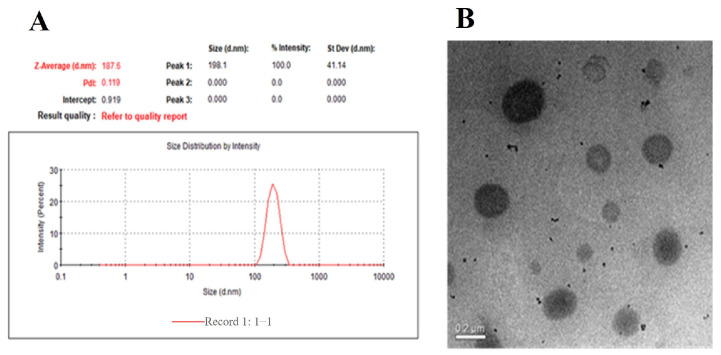
Particle size distribution of AD808 (**A**) and morphology in TEM (**B**).

The particle size, Zeta potential, polydispersity index (PDI), entrapment efficiency, and drug loading of the dual-target liposome complex AD808 are shown in Table 1. Liposomes were prepared by the ammonium sulfate gradient method, and the final drug encapsulation rate was 88.7%, which met the requirements of the experiment. The particle size of the blank liposome was 182.6 ± 0.6 nm. After the protein was linked with AD808, the particle size increased slightly to 198.1 ± 1.21 nm, and the PDI was less than 0.2, which indicated that the liposomes before and after the protein was linked were homogeneous and suitable for administration. The Zeta potential of AD808 is negative, which is beneficial to cell absorption. The protein content in the dual-target complex liposome sample was determined by the BCA method, and the protein linkage was calculated to be 231.4 ± 16.5 μg/mL. The morphology of AD808 was observed by projection electron microscope, and it still had spherical characteristics, and the particle size was relatively uniform (Figure 2).

### 2.2. Evaluation of Blood–Brain Barrier Permeability

The blood–brain barrier (BBB) is a biological barrier composed of microvascular endothelial cells, basement membrane, and astrocytes. BEnd.3 cells were selected to establish the blood–brain barrier (BBB) model and evaluated for permeability following AD808 administration in the upper chamber.

**Figure 3:**
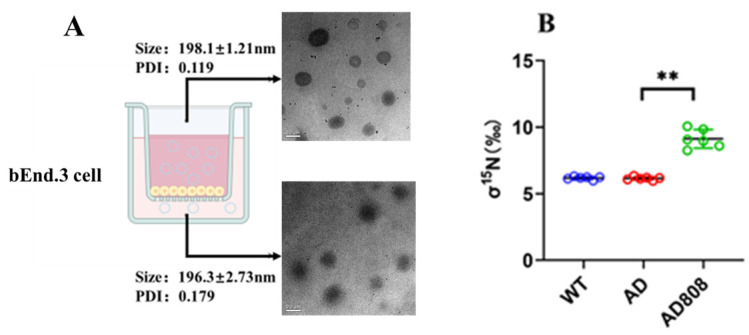
Morphology of AD808 before and after crossing the blood–brain barrier in TEM (**A**). After intravenously injection with AD808 (15N-gh625-Zn_7_MT3 labeled), notably higher 15N stable isotope abundance (**B**) was observed. Scale bar = 0.2 μm. The statistics were conducted with the ANOVA test. (The results as shown as mean ± S.D. *n* = 6, **, *p* < 0.01.)

TEM (transmission electron microscope) analysis (Figure 3) demonstrated that the dual-target liposome complex AD808 maintained its structural integrity and particle size distribution after blood–brain barrier (BBB) traversal, confirming excellent stability. Quantification of phospholipid and ST707 concentrations in both chambers revealed a 57.2% penetration rate for AD808, demonstrating effective blood–brain barrier (BBB) permeability suitable for in vivo administration.

The AD808-treated group exhibited significantly higher 15N abundance (*p* < 0.01) compared to the control AD model (Figure 3B), demonstrating AD808’s ability to cross the blood–brain barrier (BBB) in this AD mouse model.

### 2.3. AD808 Ameliorates Cognitive Deficits in AD Mice

**Figure 4:**
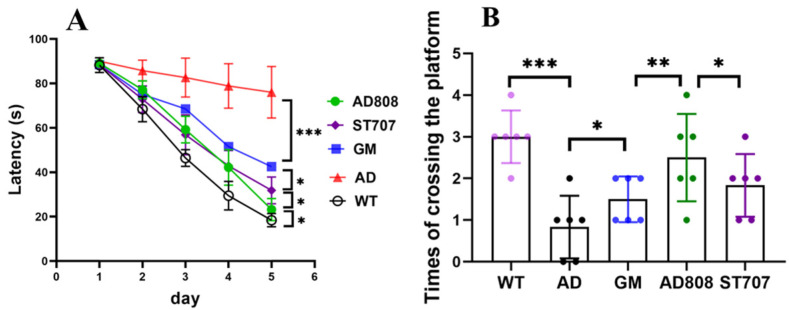
Latency of Morris water maze (**A**) and times of crossing the platform (**B**) (Two-way ANOVA test in A and one-way ANOVA test in (**B**)). (The results as shown as mean ± S.D. *n* = 6, ***, *p* < 0.001.**, *p* < 0.01. *, *p* < 0.05.)

Figure 4A shows that from day 3 of training, wild-type (WT) mice exhibited significantly shorter escape latencies than APP/PS1 mice (AD model group), confirming severe spatial learning/memory impairment in the AD model. The ST707 and gh625-Zn_7_MT3 (GM) groups showed markedly reduced escape latencies versus the AD group from day 3 (*p* < 0.01), with 59% and 45% reductions by day 5, respectively, demonstrating partial cognitive recovery. AD808 treatment reduced escape latency by 70% versus the AD group (*p* < 0.001), outperforming both monotherapies and confirming robust restoration of learning capacity. AD808’s superiority over the ST707 and GM groups (*p* < 0.05) highlights the synergistic benefits of dual-target liposomal delivery.

On the last day of the training period, the platform was removed to examine the spatial memory ability of mice. Figure 4B reveals that AD mice made 0.8 ± 0.7 platform crossings (vs. 3.2 ± 0.9 in WT; *p* < 0.001), reflecting >70% impairment. The above results showed that the spatial memory ability of the mice in the AD group was seriously damaged. The ST707 and GM groups showed improvement (1.8 ± 0.5 and 1.5 ± 0.6 crossings, *p* < 0.05). AD808-treated mice achieved 2.6 ± 0.6 crossings, which significantly improved the spatial memory ability of AD mice, significantly surpassing both the AD group (*p* < 0.001) and the monotherapy group (*p* < 0.01 vs. ST707/GM).

### 2.4. AD808 Improves the Pathomorphology of the Hippocampus in Alzheimer’s Disease (AD) Mice

**Figure 5:**
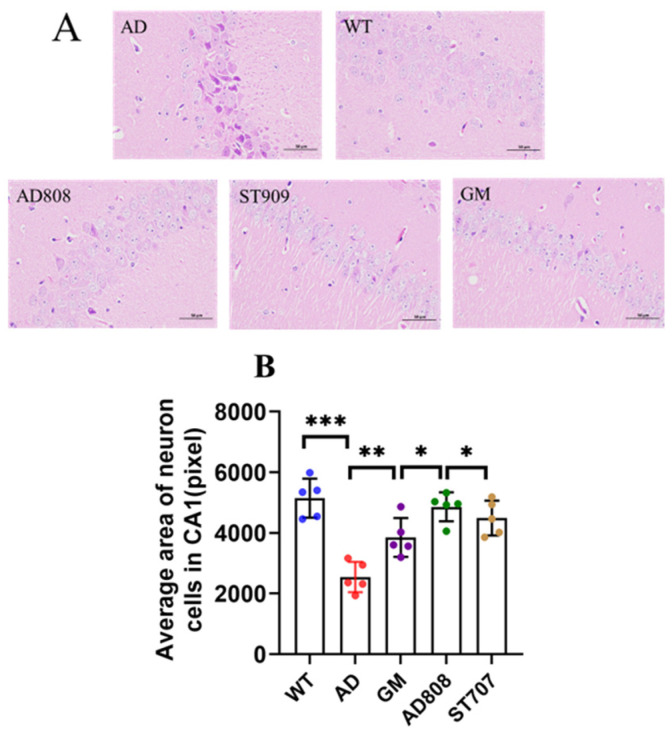
Haematoxylin–Eosin (HE) staining assay of the hippocampus CA1 region revealed the progression of neuronal apoptosis (**A**,**B**). Scale bar = 50 μm. The statistics were conducted with the ANOVA test. (The results as shown as mean ± S.D. *n* = 5, ***, *p* < 0.001.**, *p* < 0.01. *, *p* < 0.05.)

In the CA1 area of the hippocampus in wild-type (WT) mice, neurons were plump and closely arranged, resisting hematoxylin staining and thus appearing lightly stained (Figure 5A). In contrast, in the AD group, the hippocampal CA1 region exhibited significant neuronal loss, shrunken morphology, disrupted cellular structure, and disorganized arrangement, making it more susceptible to hematoxylin staining and thus appearing darker. After six weeks of treatment, neurons in the CA1 region of mice from the three experimental groups (ST707, GM, and AD808) showed significant improvement. Compared with the AD group, neuronal counts increased, morphology appeared more robust and intact, and cellular arrangement was more organized. Collectively, these findings indicate that these compounds significantly ameliorated pathological features in the CA1 region of AD mice. Statistical analysis of H&E staining (Figure 5B) revealed that AD808 exhibited approximately 105% greater staining intensity compared to the AD group.

**Figure 6:**
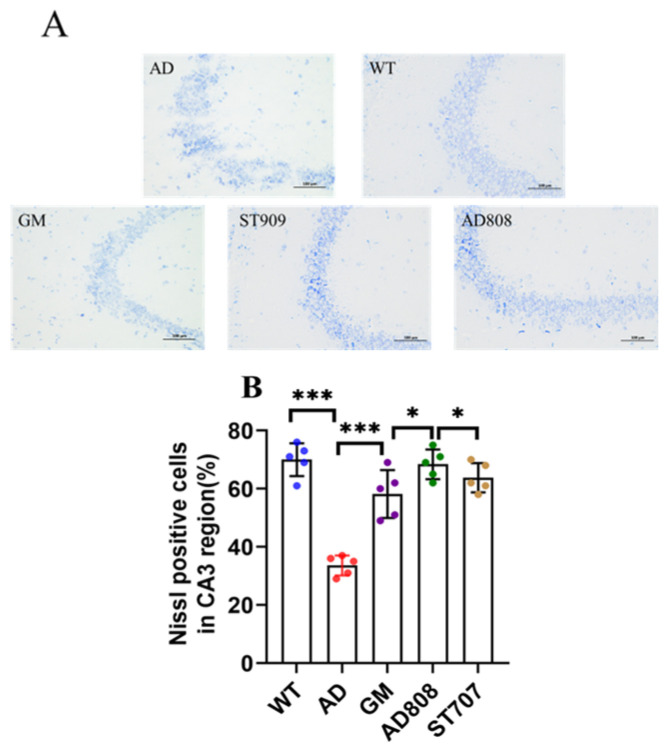
Nissl staining assay of the hippocampus CA3 region revealed the progression of neuronal damage (**A**,**B**). Scale bar = 50 μm. The statistics were conducted with the ANOVA test. (The results as shown as mean ± S.D. *n* = 5, ***, *p* < 0.001. *, *p* < 0.05.)

Nissl staining (Figure 6A) showed that Nissl corpuscles were abundant in the CA3 area of the hippocampus in wild-type (WT) mice, and their structures were intact. In contrast, in the AD group, the number of Nissl bodies decreased significantly, indicating that neuronal number and density were markedly reduced. Six weeks after treatment, the number of Nissl bodies in the CA3 area of the mouse hippocampus showed a significant increase in the three experimental groups (ST707, GM, and AD808). Compared with the AD group, neuronal number and density showed significant increases in all treatment groups. Statistical analysis of Nissl staining (Figure 6B) revealed that AD808 exhibited approximately 73% greater staining intensity compared to the AD group.

### 2.5. AD808 Reduced Neuronal Apoptosis in Alzheimer’s Disease (AD) Mice

**Figure 7:**
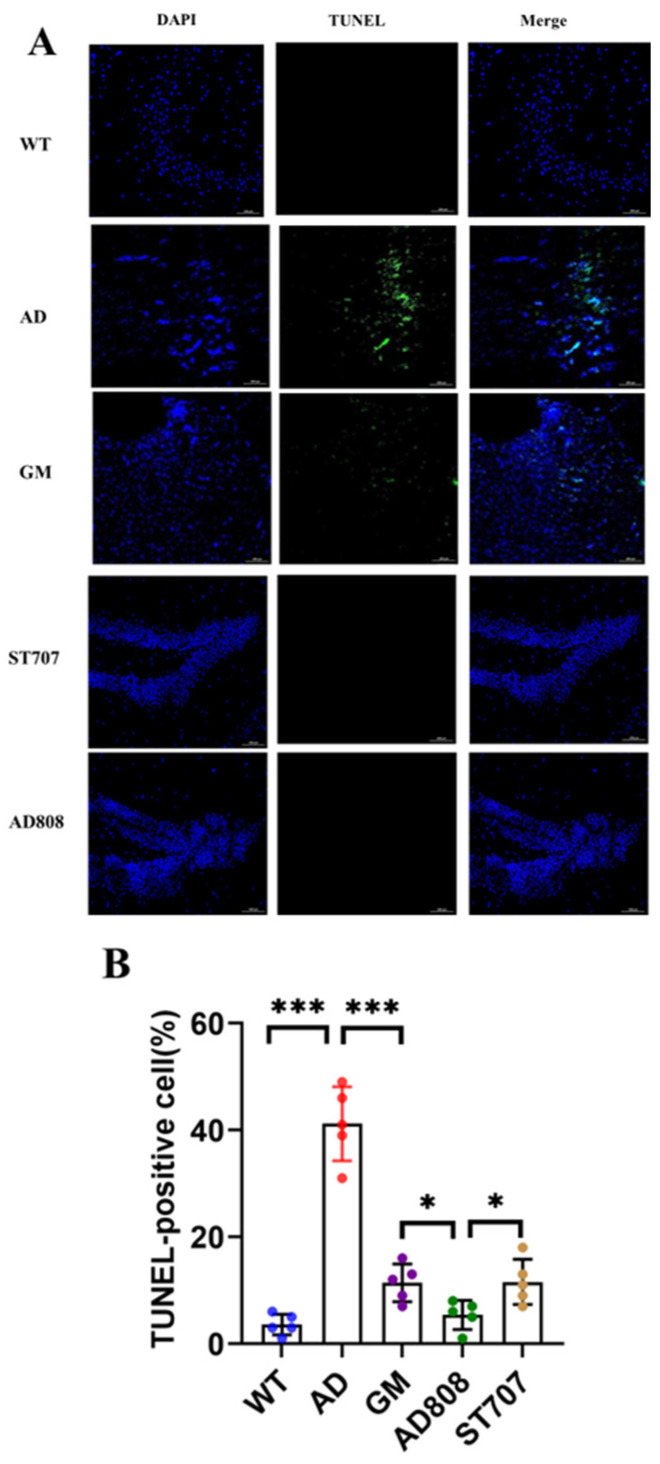
TUNEL staining (green) reflected the level of apoptosis, while DAPI staining (blue) was used as a counterstain revealing nuclei (**A**). The proportion of TUNEL-positive cells (**B**). Scale bar = 100 μm. The statistics were conducted with the ANOVA test. (The results as shown as mean ± S.D. *n* = 5, ***, *p* < 0.001. *, *p* < 0.05.)

TUNEL staining was performed to assess neuronal apoptosis, and images were acquired using a laser confocal microscope. As shown in Figure 7A, apoptotic neurons exhibited green fluorescence, whereas viable neurons were counterstained blue. Compared with wild-type (WT) controls, AD mice showed significantly enhanced green fluorescence intensity, indicating substantial neuronal apoptosis. In the GM group, fluorescence intensity was markedly reduced, demonstrating decreased neuronal apoptosis. Notably, both AD808- and ST707-treated groups exhibited minimal fluorescence signal, suggesting near-complete suppression of neuronal apoptosis. Quantitative analysis of TUNEL-positive cells (Figure 7B) confirmed these findings. AD808 treatment resulted in significantly fewer TUNEL-positive cells compared to the AD group (*p* < 0.001).

### 2.6. AD808 Reduced Amyloid Plaque Burden in Alzheimer’s Disease (AD) Mouse Brains

**Figure 8:**
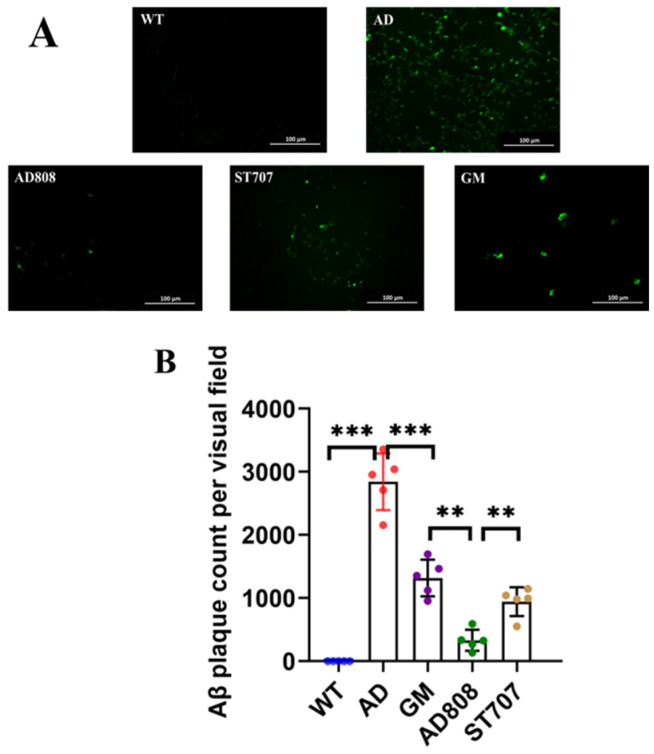
Thioflavin-S staining (green) indicated Aβ plaques aggregated in brain tissues (**A**). Quantification of numbers and surface area of Aβ plaques (**B**). Scale bar = 100 μm. The statistics were conducted with the ANOVA test. (The results as shown as mean ± S.D. *n* = 5, ***, *p* < 0.001. **, *p* < 0.01.)

Aβ deposition is a pathological hallmark of AD. Thioflavin S staining revealed Aβ aggregates exhibiting green fluorescence when visualized by confocal microscopy. As shown in Figure 8A, AD mice exhibited significantly more extensive Aβ deposition (green fluorescence), with larger plaque sizes compared to WT controls. ST707- and GM-treated mice showed markedly reduced fluorescence intensity, with only sparse plaques remaining. AD808 treatment nearly abolished fluorescence signals, suggesting >90% clearance of Aβ aggregates compared to AD. Quantitative analysis of thioflavin S staining (Figure 8B) confirmed that AD808 treatment resulted in significantly reducing Aβ plaque compared to the AD group (*p* < 0.001).

**Figure 9:**
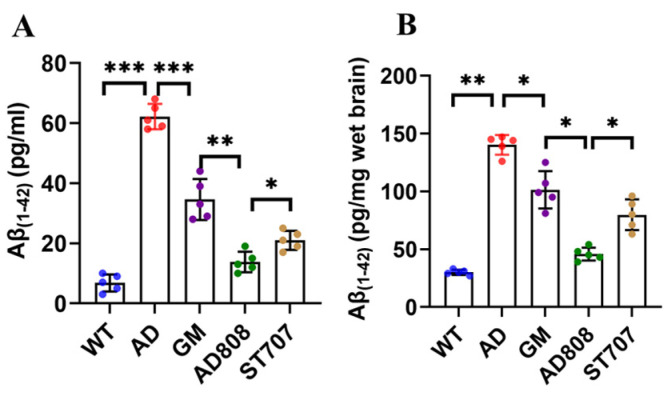
Aβ_(1-42)_ concentration in serum (**A**) and in brain issues (**B**). The statistics were conducted with the ANOVA test. (The results as shown as mean ± S.D. *n* = 5, ***, *p* < 0.001. **, *p* < 0.01. *, *p* < 0.05.)

Serum-soluble Aβ levels were quantified by ELISA (Figure 9A). AD mice showed elevated serum Aβ levels (58.2 ± 3.6 pg/mL) compared to WT (*p* < 0.001). All treatments (ST707, GM, and AD808) significantly reduced serum Aβ levels (*p* < 0.001), with reductions of 43% (GM), 54% (ST707), and 75% (AD808) relative to AD controls. Notably, AD808 showed superior Aβ clearance versus AD (*p* < 0.001), surpassing ST707- and GM-treated mice.

Quantification of Aβ levels in brain tissue by ELISA (Figure 9B) demonstrated that, consistent with the plasma-soluble Aβ results, AD808-treated mice exhibited significantly reduced Aβ1-42 levels in brain compared to the AD group (*p* < 0.01).

### 2.7. AD808 Ameliorates Neuroinflammation in Alzheimer’s Disease (AD) Mice

Chronic neuroinflammation is a well-established pathological feature of AD. Accumulating evidence indicates that hyperactivation of inflammatory signaling pathways correlates strongly with learning deficits and cognitive impairment in AD mice, where inflammatory cytokine levels serve as direct biomarkers of neuroinflammatory progression [21]. To investigate AD808’s mechanism of action, both pro-inflammatory (TNF-α, IL-1β) and anti-inflammatory (IL-4, IL-10) cytokine profiles were quantified.

**Figure 10:**
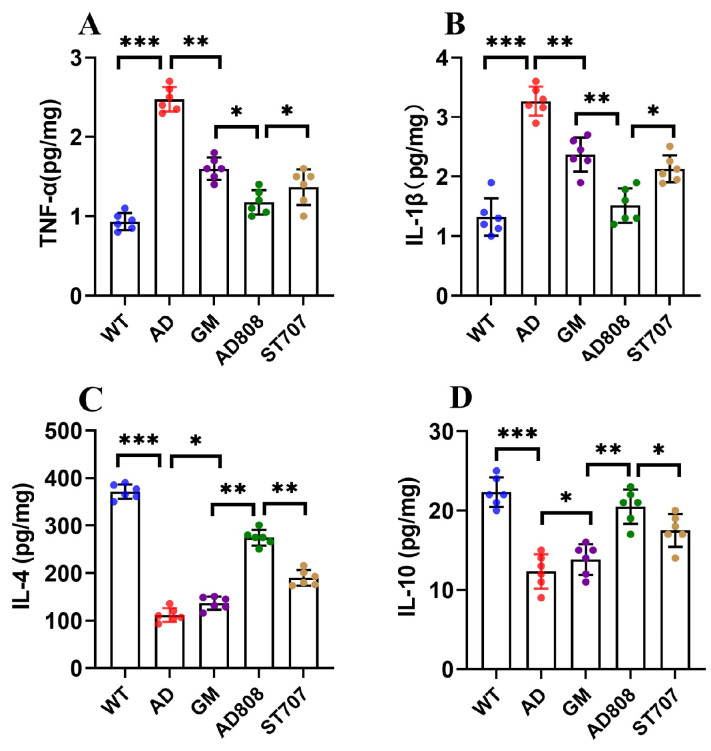
Quantification of inflammatory factors in brain tissue by ELISA. The statistics were conducted with the ANOVA test. ((**A**): TNF-α; (**B**): IL-1β; (**C**): IL-4; (**D**): IL-10. *n* = 5; the results as shown as mean ± S.D. ***, *p* < 0.001.**, *p* < 0.01. *, *p* < 0.05.)

As shown in Figure 10A,B, AD mice exhibited significant neuroinflammation. Compared to wild-type controls, TNF-α and IL-1β concentrations in AD mouse brains were elevated 2.76-fold and 2.56-fold, respectively (*p* < 0.001). The concentrations of TNF-α and IL-1β in the AD808 group were significantly lower than those in the ST707 and GM group s(*p* < 0.01). Treatment with ST707 reduced these pro-inflammatory cytokines by 40.2% (TNF-α) and 36.7% (IL-1β), while AD808 demonstrated superior efficacy, with reductions of 57.3% (TNF-α) and 61.5% (IL-1β) relative to AD controls. The above results show that AD808 can significantly reduce the level of inflammatory factors in the central nervous system.

Notably, AD mice showed significantly depleted anti-inflammatory cytokines—IL-4 (110.2 ± 25.7 pg/mg, 71.8% decrease) and IL-10 (14.6 ± 2.9 pg/mg, 44.7% decrease) compared to wild-type. Six-week treatment with ST707, GM and AD808 increased IL-4 levels by 23.6%, 61.3%, and 154.5%, and IL-10 by 28.5%, 50.2% and 101.1%, respectively. AD808’s anti-inflammatory effects were significantly enhanced versus AD group (*p* < 0.001).

These findings demonstrate that while ST707 effectively modulates neuroinflammatory responses, the dual-target liposome complex AD808 (loading ST707) provides significantly greater suppression of pro-inflammatory cytokines, restoration of anti-inflammatory factors, and consequent neuroprotection in the inflammatory microenvironment, ultimately improving cognitive function in AD mice.

### 2.8. AD808 Can Transform M1-Polarized Microglia into the M2 Phenotype

Microglia are resident macrophages in the central nervous system (CNS) and represent a major immune cell population [22]. Microglia are highly sensitive to changes in the neuronal extracellular environment, dynamically switching between the pro-inflammatory M1 and anti-inflammatory M2 phenotypes, thereby secreting cytokines that influence astrocytes and neurons. In AD mice, microglia shift from the M0 to the M1 phenotype, releasing pro-inflammatory factors, whereas the M2 phenotype secretes anti-inflammatory factors to promote tissue repair. Improper microglial activation exacerbates AD progression, as AD risk factors are closely linked to microglial dysfunction and their response to amyloid plaques [23].

**Figure 11:**
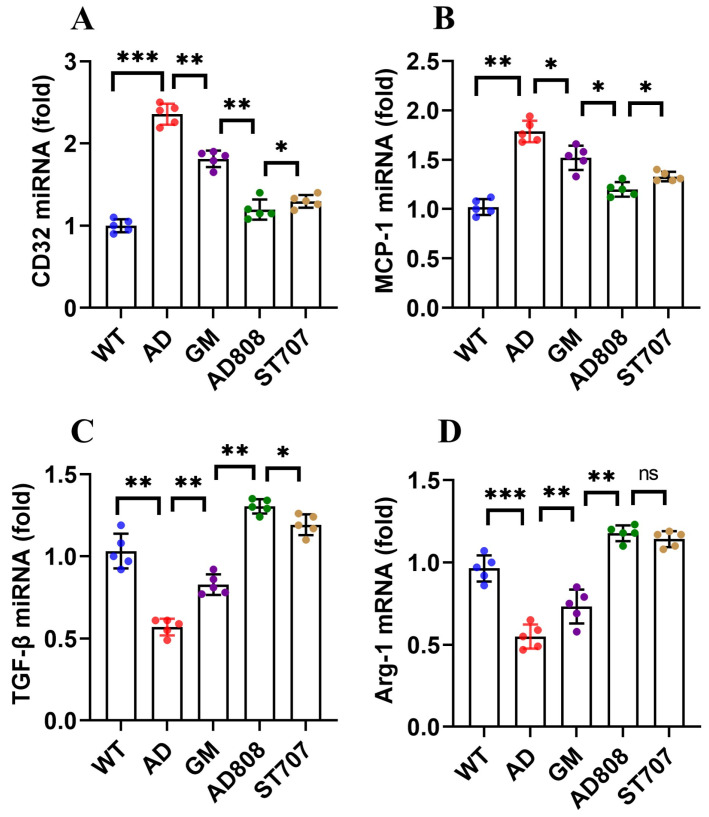
mRNA expression levels of M1 markers (CD32, MCP-1) and M2 markers (TGF-β, Arg-1) in microglia. The statistics were conducted with the ANOVA test. ((**A**): MCP-1; (**B**): CD32; (**C**): TGF-β; (**D**): Arg-1. *n* = 5; the results as shown as mean ± S.D. ***, *p* < 0.001.**, *p* < 0.01. *, *p* < 0.05. ns, *p* > 0.05).

To investigate the effects of ST707, GM, and AD808 on microglia, the mRNA levels of M1 markers (MCP-1, CD32) and M2 markers (TGF-β, Arg-1) were measured via RT-qPCR (Figure 11). Compared to the normal group, M1 markers (MCP-1, CD32) in the AD group increased by 71% and 143%, respectively, whereas M2 markers (TGF-β, Arg-1) decreased by 43% and 48%. After six weeks of treatment with ST707, GM, or AD808, microglial polarization was significantly modulated. In the AD808 group, MCP-1 and CD32 levels decreased by 32% and 50%, respectively, while M2 markers (TGF-β, Arg-1) increased by 136% and 118%, outperforming ST707 and GM. Compared to the AD group, AD808 treatment significantly upregulated expression of M2 markers (TGF-β, Arg-1) while suppressing M1 markers (MCP-1, CD32) (*p* < 0.001).

The dual-target liposome AD808, via sustained release and precise delivery, potentiated ST707’s effects for suppressing Aβ-induced neuroinflammation, promoting microglial polarization from the M1 to M2 phenotype, reducing apoptosis, and improving cognitive function in AD mice.

### 2.9. AD808 Promotes the Expression of Neurotrophic Factors in the Brain Tissue of Alzheimer’s Disease (AD) Mice

Based on these findings, it was concluded that ST707 can induce microglial polarization toward the M2 phenotype. Microglia release various neurotrophic factors that facilitate neuronal repair and neural structure regeneration during post-injury recovery [24,25]. These results suggest that AD808’s neurorestorative effects in AD mice are mediated through microglia-derived neurotrophic factors. Brain-derived neurotrophic factor (BDNF), the most abundant neurotrophic factor in the brain, promotes neurogenesis, neuronal differentiation, and functional recovery of damaged neurons. The glial cell-derived neurotrophic factor (GDNF) protects mature neurons, promotes their repair, and enhances differentiation. Nerve growth factor-1 (NGF-1) supports sympathetic and cholinergic neurons by stimulating synaptic growth, enhancing repair, and preventing neuronal degeneration.

**Figure 12:**
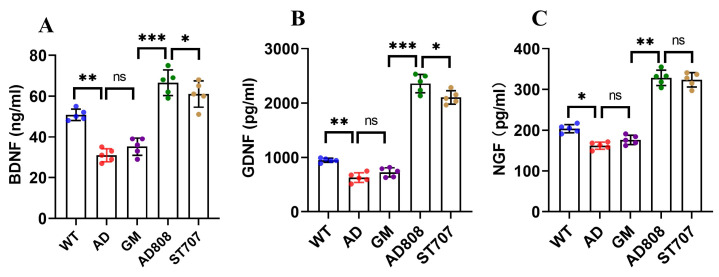
Neurotrophic factor levels in serum. The statistics were conducted with the ANOVA test. ((**A**): BDNF; (**B**): GDNF; (**C**): NGF. *n* = 5; the results as shown as mean ± S.D. ***, *p* < 0.001. **, *p* < 0.01. *, *p* < 0.05. ns, *p* > 0.05).

Neurotrophic factor levels in brain tissue were quantified by ELISA (Figure 12). Compared to wild-type (WT) controls, BDNF, GDNF, and NGF-1 levels in the AD group decreased by 30.2%, 29.6%, and 19.1%, respectively. Both ST707 and AD808 treatment significantly elevated all three neurotrophic factors (*p* < 0.001), whereas GM treatment showed no significant effect (*p* > 0.05). Relative to the AD group, AD808 treatment increased BDNF, GDNF, and NGF-1 levels by 142.8%, 275.9%, and 111.3% (*p* < 0.001). These findings demonstrate that AD808 promotes neuronal repair by shifting microglial polarization from M1 to M2 phenotype and enhancing neurotrophic factor expression. Furthermore, AD808’s sustained-release properties and blood–brain barrier targeting capability potentiate ST707’s therapeutic effects.

### 2.10. AD808 Increases MT3 Protein Levels in Brain Tissue

Metallothionein-3 (MT3) regulates metal homeostasis in the brain and participates in various physiological processes, including zinc-metalloprotein synthesis and extracellular heavy metal detoxification [12,26]. Studies indicate a 30% reduction in MT3 protein levels in AD patients’ brains, resulting in disrupted metal homeostasis, copper (Cu) overload, and exacerbated oxidative stress [27,28]. Therefore, restoring MT3 levels may ameliorate metal homeostasis imbalance in AD mouse brains.

**Figure 13:**
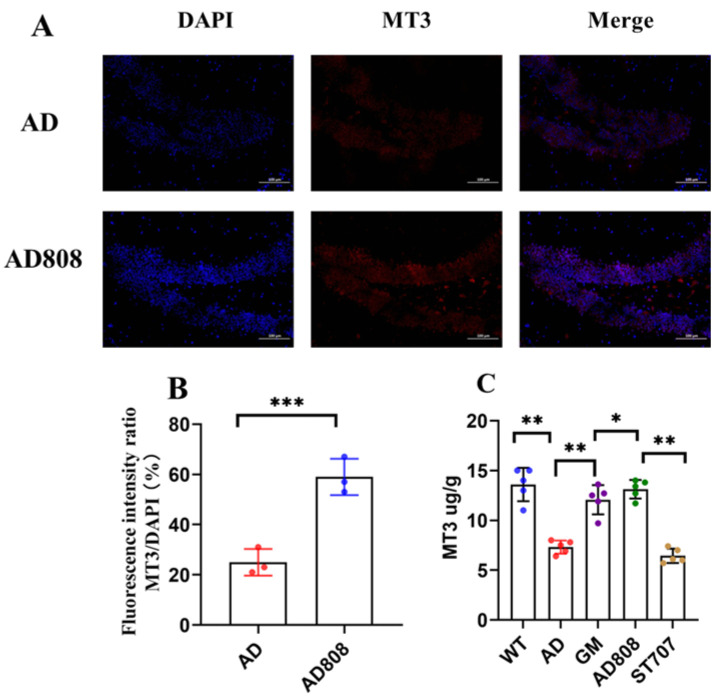
Regional distribution of MT3 in brain tissue (**A**), statistical results of fluorescence intensity (*n* = 3) (**B**), and concentration of MT3 in brain tissues (**C**) (*n* = 5). Scale bar = 100 µm. The statistics were conducted with the ANOVA test. (The results as shown as mean ± S.D. ***, *p* < 0.001. **, *p* < 0.01. *, *p* < 0.05.)

MT3 protein in brain tissue was labeled by immunofluorescence and imaged using confocal microscopy (Figure 13A). Following 6-week AD808 treatment, MT3 immunofluorescence signals (red) were markedly enhanced, demonstrating upregulated MT3 concentration in the hippocampus that may ameliorate AD progression. These findings support that AD808 effectively replenishes MT3 in neural tissue.

Additionally, MT3 levels were quantified by ELISA to compare AD808’s efficacy with ST707 and GM treatments (Figure 13C). The AD808 group showed MT3 concentrations of 13.8 ± 1.2 μg/g, representing a 16.2% increase over ST707. Relative to gh625-Zn_7_MT3 controls, AD808-treated mice exhibited a 22.5% increase in brain MT3 content. Compared to the AD group, the AD808-treated group represents a 48.6% increase in brain MT3 content. These results suggest that AD808’s sustained-release formulation and targeted delivery minimize systemic MT3 metabolism, enhance blood–brain barrier penetration, improve metal homeostasis regulation, and reduce oxidative damage in AD mice.

### 2.11. AD808 Upregulates TREM2 Expression in Brain Tissue

TREM2, a microglia-specific surface receptor, has been identified as a genetic risk factor for AD, with reduced function correlating with disease susceptibility [17]. Notably, TREM2 expression is upregulated around Aβ plaques, suggesting a compensatory neuroprotective response during AD pathogenesis [29,30]. AD mouse models demonstrate that TREM2 knockout or mutation exacerbates neuronal damage and Aβ accumulation [30]. TREM2 activation enhances microglial Aβ clearance capacity, thereby conferring neuroprotection.

**Figure 14:**
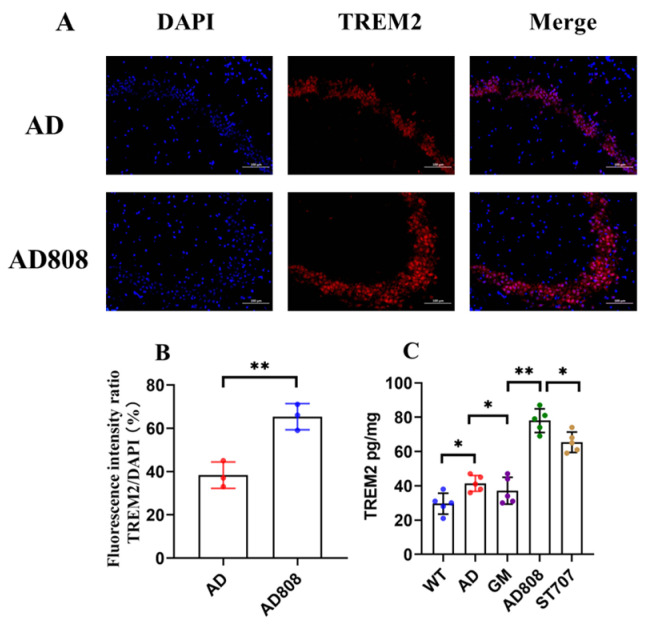
Regional distribution of TREM2 in brain tissue (**A**), statistical results of fluorescence intensity (*n* = 3) (**B**), and concentration of TREM2 in brain tissues (**C**) (*n* = 5). Scale bar = 100 μm. The statistics were conducted with the ANOVA test. (The results as shown as mean ± S.D. **, *p* < 0.01. *, *p* < 0.05.)

To assess AD808’s effects on TREM2 expression, brain sections were immunostained for TREM2 and analyzed by confocal microscopy. AD808 treatment for 6 weeks markedly increased TREM2 immunofluorescence signals in the hippocampus (Figure 14A), suggesting its potential to modulate AD progression through TREM2 upregulation. These findings support our hypothesis and were corroborated by fluorescence intensity quantification.

Quantitative ELISA revealed significantly greater TREM2 upregulation by AD808 compared to AD (Figure 14C). The AD808 group exhibited brain TREM2 levels of 87.2 ± 5.4 pg/mg, representing a 113.5% increase over AD mice. These results demonstrate that AD808’s sustained-release and targeted delivery properties potentiate ST707’s effects on TREM2 expression, enhancing Aβ clearance and mitigating neuroinflammation.

### 2.12. AD808-Mediated Neural Repair via the STING/IRF3 and PI3K/AKT Pathways

**Figure 15:**
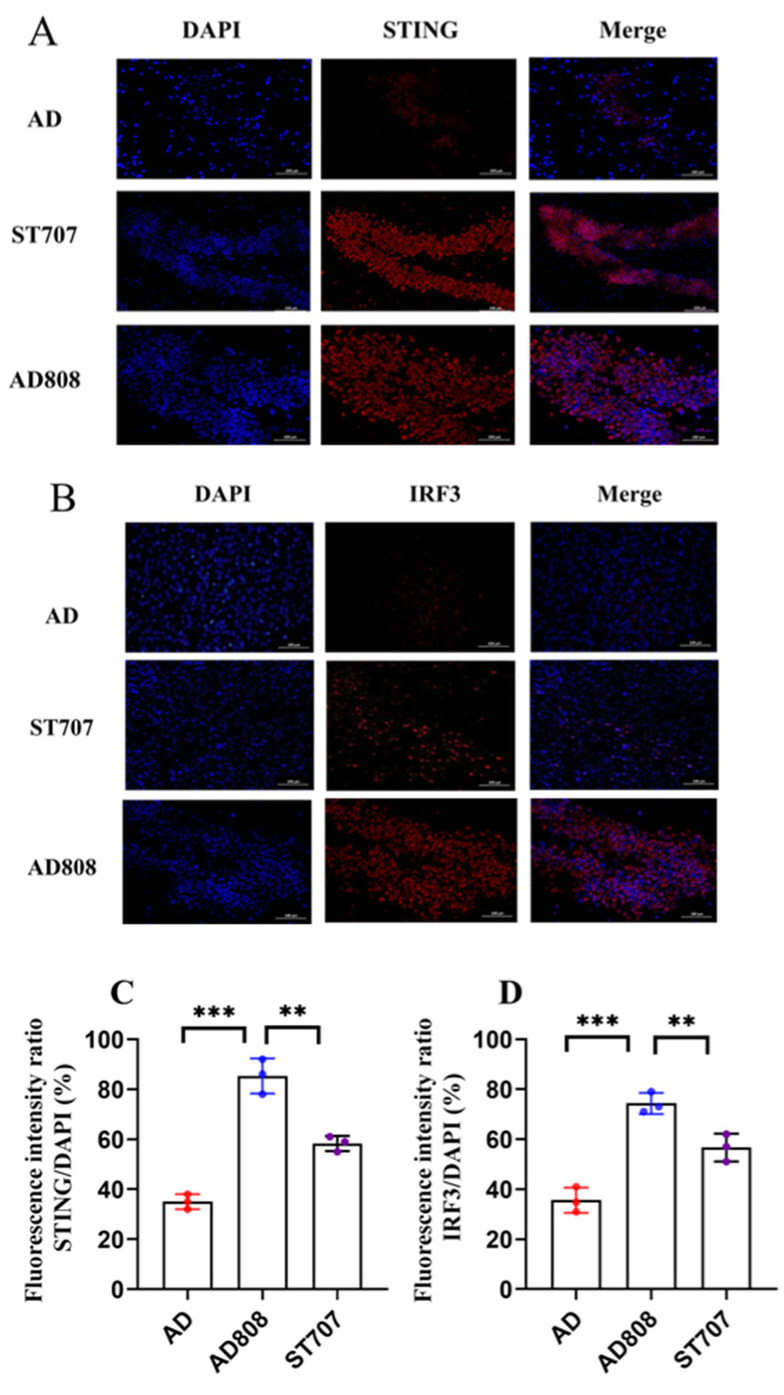
ST707 induces activation of the STING/IRF3 pathway, as demonstrated by immunofluorescence (**A**,**B**). Scale bar = 100 μm. The statistics were conducted with the ANOVA test. Quantitative analysis was presented as mean ± SEM ((**C**,**D**), *n* = 3, **, *p* < 0.01. ***, *p* < 0.001).

Previous studies have demonstrated that ST707 functions as a secondary messenger to activate the cGAS-cGAMP-STING-IRF3 signaling pathway, triggering innate immune responses. To elucidate ST707’s mechanism of action, STING/IRF3 pathway activation was assessed using immunofluorescence staining. In Figure 15A,B, target proteins (red) and nuclei (blue) were visualized by immunofluorescence. Fluorescence intensity and puncta density positively correlated with target protein expression levels. Quantitative analysis revealed significantly lower STING and IRF3 expression in the AD group compared to controls. Both ST707- and AD808-treated groups exhibited significant upregulation of STING and IRF3 compared to the AD group. Semi-quantitative analysis of fluorescence intensity was performed (Figure 15C,D). Relative to the AD group, STING fluorescence intensity increased by 63% in ST707-treated mice and 137% in AD808-treated mice. Similarly, IRF3 intensity increased by 132% (AD808) and 67% (ST707), with AD808 showing significantly greater efficacy (*p* < 0.01). Compared to AD, AD808 significantly increased STING (115%) and IRF (85%) (*p* < 0.001).

These findings demonstrate that AD808 activates STING, leading to IRF3 phosphorylation and nuclear translocation, thereby initiating type I interferon responses. AD808’s sustained-release formulation and blood–brain barrier targeting capability significantly enhance ST707’s immunomodulatory effects.

**Figure 16:**
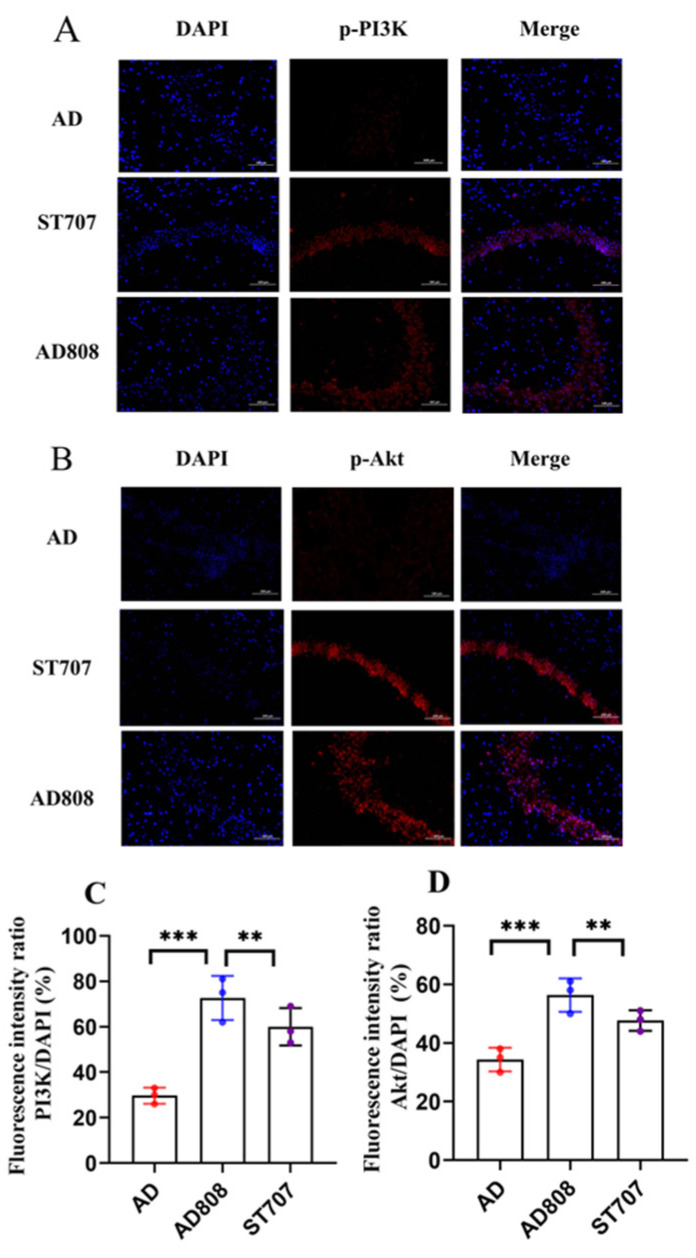
AD808 induces activation of the PI3K/AKT pathway, as demonstrated by immunofluorescence (**A**,**B**). Scale bar = 100 μm. The statistics were conducted with the ANOVA test. Quantitative analysis was presented as mean ± SEM ((**C**,**D**), *n* = 3, **, *p* < 0.01. ***, *p* < 0.001).

The PI3K/AKT/GSK-3β signaling pathway has been implicated in Alzheimer’s disease pathogenesis. In AD, reduced PI3K phosphorylation attenuates AKT activation, resulting in GSK-3β hyperactivity that drives tau hyperphosphorylation and Aβ deposition [31]. Additionally, PI3K/AKT activation promotes microglial polarization from pro-inflammatory M1 to anti-inflammatory M2 phenotype, mitigating neuroinflammation [19]. Therefore, this mechanism was investigated using immunofluorescence analysis. Figure 16A,B illustrate target protein (red) and nuclear (blue) staining patterns. Fluorescence intensity and puncta density correlated with target protein expression levels. Quantitative analysis revealed significantly lower p-PI3K and p-AKT expression in AD mice versus controls. Both ST707 and AD808 treatment significantly increased p-PI3K and p-AKT levels compared to the AD group. Semi-quantitative analysis of fluorescence intensity was performed (Figure 15C,D). Relative to the AD group, p-PI3K fluorescence intensity increased by 93% (ST707) and 151% (AD808) (*p* < 0.01), while p-AKT increased by 37% (ST707) and 65% (AD808) (*p* < 0.01). AD808 demonstrated significantly greater efficacy than AD (*p* < 0.001).

The above results show that AD808 can further promote the activation of PI3K in an inflammatory environment and induce the phosphorylation of AKT through the STING protein. On the one hand, it upregulates the expression of anti-apoptotic Bcl-2 protein in cells, suppresses Aβ synthesis, and inhibits apoptosis [32]. On the other hand, it enhances AKT phosphorylation, thereby inhibiting GSK-3β activity and reducing tau protein hyperphosphorylation [33].

## 3. Discussion

### Pharmacological Mechanism of AD808 in the Treatment of Alzheimer’s Disease

Alzheimer’s disease (AD) is a common neurodegenerative disease associated with aging. The extracellular accumulation of amyloid plaques and intracellular neurofibrillary tangles formed by hyperphosphorylated tau protein are considered the hallmark pathological features of AD. In AD, microglia exhibit dual roles, exerting both neuroprotective and neurotoxic effects [34]. Amyloid plaques accumulated extracellularly can trigger an immune response, activating microglia and astrocytes to release proinflammatory factors, chemokines, and neurotoxins via NF-κB, thereby activating apoptotic pathways and severely disrupting neuronal metabolism [14]. Studies indicate that microglia are key drivers of neuroinflammation and neuronal apoptosis in AD progression. Conversely, microglia can phagocytose Aβ oligomers and damaged cells, while promoting Aβ clearance via proteases such as insulin-degrading enzyme (IDE) and matrix metalloproteinase-9 (MMP-9) [29]. Recent studies confirm that microglia polarize into pro-inflammatory M1 and anti-inflammatory M2 phenotypes during immune responses. M2 microglia secrete anti-inflammatory factors, while TREM2-mediated phagocytosis of Aβ plaques contributes to neuroprotection and repair [35]. Therefore, immunomodulation and anti-inflammatory therapy based on microglia are important targets for the treatment of Alzheimer’s disease.

TREM2, a microglial surface receptor, is genetically linked to AD risk [17]. TREM2 deficiency promotes Aβ deposition and neuronal apoptosis [29]. Related studies have shown that the process of microglia removing Aβ amyloid plaques depends on the mechanism of TREM2, so increased TREM2 expression helps reduce Aβ amyloid plaques and maintain microglial clearance [15]. Xu et al. demonstrated that cGAMP, an innate immune signaling molecule, upregulated TREM2 expression in microglia via STING/IRF3 activation [20]. In the APP/PS1 mouse model, the upregulated expression of TREM2 protein effectively cleared Aβ amyloid plaques in the brain, improved spatial cognitive impairment in mice, and inhibited neuroinflammation and neuronal apoptosis [20]. Jiang et al. also found that in the APP/PS1 mouse model, loss of TREM2 function impaired microglial Aβ clearance. By upregulating TREM2 expression, the TREM2-DAP12 complex in microglia bound Aβ and transmitted intracellular signals via protein tyrosine kinase (SYK), activating microglial clearance [18,36].

Accumulating evidence indicates that AD pathogenesis involves disrupted metal homeostasis and aberrant metalloprotein expression. In the brain microenvironment of AD patients, Cu^2+^ overload and Zn^2+^ deficiency were observed, and MT3 protein regulated Cu/Zn metabolism [28]. Xu et al. showed that Zn_7_MT3 restores zinc homeostasis in the brain microenvironment and chelates excess Cu^2+^ to inhibit Cu-induced Aβ aggregation, mitigating Aβ neurotoxicity [13]. Using the APP/PS1 mouse model, Xu et al. confirmed that Zn_7_MT3 significantly improves spatial cognitive impairment, reduces hippocampal neuronal apoptosis, and alleviates neuropathological damage [13]. Manso et al. demonstrated that exogenous MT3 administration increased soluble Aβ40 levels, reduced insoluble amyloid plaque formation, and improved behavioral deficits in AD mice [27]. These findings suggest that TREM2 protein and MT3 protein are important therapeutic targets for AD.

In this study, to enhance the anti-AD effects of the innate immunomodulator ST707 and the brain transition metal homeostasis-regulating protein Zn_7_MT3, a dual-target liposome complex was designed and prepared for combined immunomodulation and metal homeostasis regulation. gh625, which conjugated to Zn_7_MT3, is a synthetic peptide derived from the HIV-1 gp41 glycoprotein, and belongs to the cell-penetrating peptide (CPP) family [37]. Due to its ability to efficiently traverse cell membranes, it holds significant promise as a drug delivery vehicle [38]. Studies indicate that gh625 could cross the blood–brain barrier (BBB), suggesting its potential for delivering therapeutics to the central nervous system (CNS) to treat neurodegenerative disorders such as Alzheimer’s and Parkinson’s diseases [39,40]. This liposome system facilitates sustained drug release and enhances blood–brain barrier penetration. Using the APP/PS1 transgenic mouse model, the pharmacodynamics and pharmacological mechanism of AD808 were investigated in AD. Our results show that AD808 treatment effectively halts AD progression, with efficacy significantly better than that of either ST707 or gh625-Zn_7_MT3 alone. In the Morris water maze test, AD808 restored learning and memory in AD mice. Plasma-soluble Aβ levels and thioflavin S staining revealed that AD808 reduced Aβ plaque burden. TUNEL, H&E, and Nissl staining demonstrated that AD808 mitigated hippocampal neuronal apoptosis and neuropathological damage, indicating improved behavioral and pathophysiological outcomes.

The remarkable therapeutic efficacy of AD808 in preventing Alzheimer’s disease (AD) progression in mice prompted further investigation into its mechanism. Based on our experimental data, the mechanism of AD808—a dual-target liposome complex—can be summarized as follows. The gh625-Zn_7_MT3 protein conjugated to AD808 facilitates blood–brain barrier targeting and enables co-delivery of ST707 and recombinant gh625-Zn_7_MT3. AD808 exhibits significantly enhanced therapeutic effects compared to ST707 alone, primarily due to the synergistic interaction between the metal homeostasis regulator gh625-Zn_7_MT3 and the immunomodulator ST707.

**Figure 17:**
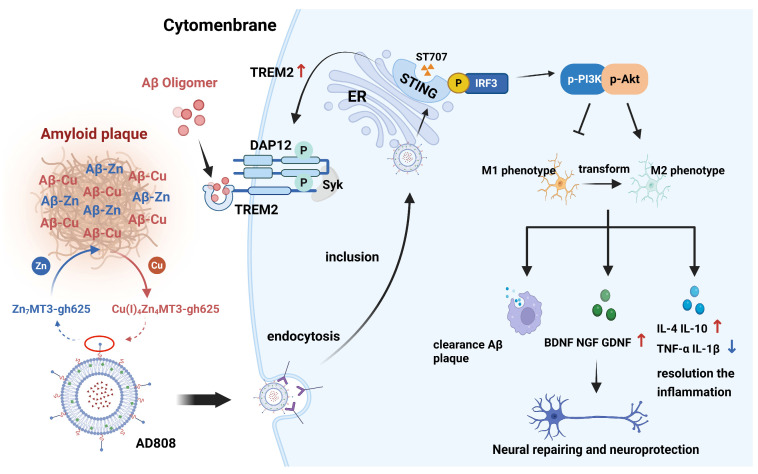
Therapeutic mechanism of AD808 in the mouse model of Alzheimer’s disease. The superior therapeutic effect of AD808 stems from the synergistic action of ST707 and gh625-Zn_7_MT3. gh625-Zn_7_MT3 regulates metal homeostasis by chelating excess Cu^2+^, reducing Cu(II) to Cu(I) and forming a stable non-toxic gh625-Cu(I)_4_Zn_4_MT3 complex, inhibiting Aβ aggregation. Cu-bound MT3 is metabolized in ER, restoring brain homeostasis. ST707 activates the STING/IRF3 pathway, upregulating TREM2, which binds DAP12 to enhance Aβ clearance and neuroprotection. AD808 activates the STING/IRF3 pathway, upregulating TREM2, which binds DAP12 to enhance Aβ clearance and neuroprotection. Phosphorylated IRF3 also activates the PI3K/AKT pathway under hypoxia, regulating the balance of M1/M2 ratios and promoting microglial polarization toward the anti-inflammatory M2 phenotype. M2 microglia secrete anti-inflammatory cytokine (e.g., IL-4, IL-10), suppressing inflammatory responses, and neurotrophic factor production (e.g., BDNF, GDNF) enhancing axonal regeneration and facilitating the repair and functional remodeling of injured neurons. Figure Created in BioRender (Premium). (https://app.biorender.com/illustrations/682a852af77e4e38e90dc000?slideId=a44ed1b1-eb3a-4e04-bc9b-8d7ba86332b8, accessed on 30 March 2025).

Upon systemic administration, the gh625-Zn_7_MT3-functionalized AD808 complex demonstrates enhanced blood–brain barrier (BBB) penetrability, enabling effective delivery to the CNS microenvironment. gh625-Zn_7_MT3, which is conjugated to AD808, acts as a redox silencer by sequestering copper and preventing its participation in ROS generation [41]. When AD808 enters the CNS microenvironment, the conjugated metallothionein-3 (gh625-Zn_7_MT3) protects neurons from the toxic effects of amyloid-β (Aβ) by facilitating a metal swap. gh625-Zn_7_MT3 removes copper (Cu) from Aβ-Cu(II) complexes, reducing Cu(II) to Cu(I) and forming a stable and non-toxic gh625-Cu(I)_4_Zn_4_MT3 complex [42]. This process abolishes reactive oxygen species (ROS) production and associated cellular toxicity [43]. gh625-Zn_7_MT3 can regulate the metal homeostasis by complexing the overloaded Cu metal ions in the brain, reducing the chronic toxicity caused by Cu accumulation in the brain, and inhibiting the synthesis of Aβ. Cu-bound MT3 protein is transferred into the endoplasmic reticulum and metabolized, thus regulating the homeostasis in the brain (Figure 17). gh625-Zn_7_MT3 protein can also promote the disproportionation of superoxide, effectively remove reactive oxygen species and free radicals in the brain environment, and reduce oxidative damage [13].

Microglia are a key AD therapeutic target. Pro-inflammatory M1 microglia exacerbate neuroinflammation, whereas anti-inflammatory M2 microglia promote repair. AD808 activates the immune response of microglia by activating the STING/IRF3 pathway (Figure 17). The specific receptor DAP12 in microglia will combine with the upregulated TREM2 cells to form a complex, and then regulate the anti-inflammatory response of microglia [44]. AD808 regulates the overexpression of TREM2 through microglia, and TRME2 can effectively reduce Aβ amyloid plaques in the brain, thus protecting the nervous system. Moreover, phosphorylated IRF3 can activate the PI3K/AKT pathway; Pl3K can promote the conversion of phosphoinositide diphosphate to triphosphate, recruit AKT and phosphorylate it, promote the transformation of microglia from pro-inflammatory phenotype M1 to anti-inflammatory phenotype M2, release anti-inflammatory factors such as IL-4 and IL-10, and eliminate chronic inflammation [45]. Furthermore, transformed M2 microglia will release neurotrophic factors such as BDNF and GDNF to repair damaged neurons [46]. Thus, AD808 combats neuroinflammation, promotes neuronal repair, and inhibits AD progression via innate immune modulation.

In this study, the APP/PS1 mouse model was used to evaluate AD808. However, since murine models incompletely replicate human AD pathology, future studies should employ non-human primate models of Alzheimer’s disease for further validation. The liposome-based co-delivery of ST707 and gh625-Zn_7_MT3 demonstrates synergistic efficacy, offering a novel strategy for AD drug development.

The clinical translation of liposomal drug delivery systems is significantly hindered by stability issues and off-target effects. Even minimally PEGylated liposomes (e.g., irinotecan-loaded formulations) can be recognized by anti-PEG antibodies, triggering complement activation, premature drug release, and immune-mediated clearance—ultimately compromising therapeutic efficacy. Furthermore, long-term storage may lead to drug leakage, particle aggregation, or size expansion, resulting in batch-to-batch variability and elevated risks of clinical failure. Targeting peptides such as transferrin receptor ligands face additional challenges, including protease-mediated degradation and nonspecific IgM binding, which accelerate systemic clearance. These phenomena not only reduce targeting precision but may also provoke unintended immune responses. Collectively, such limitations can diminish drug delivery efficiency, exacerbate toxicity, and undermine treatment outcomes.

Despite being significant challenges for clinical translation, liposome stability and off-target effects can be effectively mitigated through advanced material optimization strategies (e.g., non-PEGylated long-circulating formulations) and refined targeting modifications (e.g., engineered stable peptides). To facilitate broader clinical adoption, future investigations should prioritize that comprehensive immunogenicity profiling in preclinical models and the development of robust, scalable manufacturing protocols for liposomal therapeutics.

## 4. Materials and Methods

### 4.1. Materials

ST707 (cyclic dinucleotide) was provided by Hangzhou Orenstar Biomed Co., Ltd. (Hangzhou, China). Traut’s reagent was purchased from Sangon Biotech (Shanghai, China) Co., Ltd. HSPC, DSPE-PEG2000, cholesterol (CHO), and maleimide-PEG2000-DSPE (PEG-Mal) were purchased from Ruixi Biotech (Xi’an, China) Co., Ltd.

### 4.2. Preparation of AD808 Liposomal Complex

The lipid film was prepared by dissolving HSPC, DSPE-PEG2000, cholesterol (CHO), and maleimide-PEG2000-DSPE (PEG-Mal) at a fit molar ratio in chloroform at 40 °C. The organic solution was transferred to a rotary evaporator flask and evaporated at 115 rpm under reduced pressure (20 Pa) to form a thin lipid film on the flask wall. The lipid film was hydrated with 250 mM ammonium sulfate solution (pH 5.4) at 60 °C for 1 h, forming multilamellar vesicles (MLVs). The suspension was extruded through polycarbonate membranes (200 nm pore size) using a lipid extruder under nitrogen pressure (500 psi) for 9 cycles to obtain unilamellar vesicles with homogeneous size distribution.

For drug loading, ST707 (drug-to-lipid ratio 1:10, *w*/*w*) was added, and the pH was adjusted to 7.4 with 0.1 M NaOH, followed by incubation at 60 °C for 1 h with gentle stirring. The liposome suspension was dialyzed against 5% glucose solution with two buffer exchanges to remove unencapsulated drug. For protein conjugation, the gh625-Zn_7_MT3 fusion protein was thiolated using Traut’s reagent (20:1 molar ratio) with gentle agitation at 37 °C for 30 min, followed by dark incubation for 1 h at room temperature, and dialyzed against EDTA-containing PBS (10 mM, pH 7.4). The thiolated protein was conjugated to maleimide-bearing liposomes at a 1:50 protein-to-lipid molar ratio with 1 mM sodium dithionite as antioxidant. After 12 h conjugation at RT, unbound proteins were removed by dialyzed against 5% glucose solution.

The liposomal complex AD808 mixed with 10% (*w*/*v*) trehalose as cryoprotectant, subjected to lyophilization, and stored at −20 °C until use.

### 4.3. Characterization of AD808 Liposomal Complex

The lyophilized liposome powder was reconstituted in PBS (pH 7.4) to the original volume. For drug quantification, the suspension was aliquoted into three 100 μL samples, each mixed with 400 μL methanol/isopropanol (1:1, *v*/*v*) as demulsifier. After vortex-mixing for 30 s at maximum speed, samples were centrifuged at 12,000× *g* for 1 min at 4 °C. The supernatant was analyzed by HPLC with UV detection at 260 nm. ST707 concentration was quantified using a standard curve (0.1–100 μg/mL, R^2^ > 0.99) established with authentic ST707.

Encapsulation efficiency (EE) and drug loading (DL) were calculated as:

$$\mathrm{EE}\;(\%)=\frac{\mathrm{ST}707_{liposomes}}{\mathrm{ST}707_{total}}\times 100\%$$

$$\mathrm{LE}\;(\%)=\frac{{\mathrm{Mass}}_{ST707}}{{\mathrm{Mass}}_{liposomes}}\times 100\%$$

where:

ST707*_liposomes_* = drug (ST707) amount in liposomes (μg)

ST707*_total_* = total input drug (ST707) amount in loading process (μg)

Mass*_ST_*_707_ = mass of drug (ST707) in liposomes (mg)

Mass*_liposomes_* = total lipid mass (mg)

A certain volume of liposomes was added to 0.4 mL of methanol solution and 0.2 mL of dichloromethane, followed by 0.1 mL of water and vortex-mixing. The mixture was centrifuged at 9000 g for 1 min. After adding 0.3 mL methanol to the organic layer and vortex-mixing, the sample was recentrifuged (9000× *g*, 1 min). The supernatant was discarded to collect the protein precipitate. Residual solvent was evaporated under nitrogen stream, and the pellet was resuspended in 200 μL buffer (20 mM HEPES, 140 mM NaCl, 2% SDS). Protein concentration was determined using BCA assay with bovine serum albumin as standard.

Following calibration of a zeta potential/particle size analyzer (Malvern Zeta sizer Nano ZS), 1.0–1.5 mL liposome suspension was loaded into a quartz cuvette after rinsing with distilled water. Measurements included hydrodynamic diameter (by dynamic light scattering), polydispersity index (PDI), zeta potential (laser Doppler velocimetry).

### 4.4. Establishment of In Vitro Blood–Brain Barrier Model and Evaluation of AD808 Permeability

bEnd.3 mouse brain endothelial cells were maintained in DMEM (10% FBS, 1% penicillin/streptomycin) at 37 °C/5% CO_2_. At 90% confluence, cells were seeded (5 × 10^5^ cells/insert) onto collagen-coated Transwell^®^ inserts and cultured for 7 days with medium changes every 48 h. Model integrity was validated by TEER (Transepithelial Electrical Resistance) measurements (210 Ω·cm^2^) [47]. Ag/AgCl electrodes were vertically inserted into both the apical and basolateral compartments of the Transwell chamber. A low-amplitude alternating current (AC) was applied to prevent electrode polarization, and the resultant resistance (Ω) was recorded, with higher values indicating greater barrier integrity.

TEER were calculated as:

TEER (Ω⋅cm^2^) = (R*_sample_* − R*_blank_*) × A

where:

R*_sample_*: Cell layer resistance

R*_blank_*: Cell free spatiotemporal white resistance (medium + membrane only)

A: membrane surface area

Liposomes were added to the apical chamber, with D-Hanks’ balanced salt solution in the basolateral compartment. After 4 h incubation at 37 °C, samples from both chambers were fixed (2.5% glutaraldehyde) for TEM observation. ST707 content was analyzed by HPLC. Phospholipid concentration was determined by Stewart assay [48]. Penetration rate was calculated as (C_above_/C_bottom_) × 100%

### 4.5. 15N Stable Isotope Ratio Analysis

Stable isotope tracing method was adopted to verify the ability of AD808 to penetrate the blood–brain barrier (BBB), each AD mouse was intravenously injected with 200 μL AD808 (15N-gh625-Zn_7_MT3 labeled) and sacrificed after 4 hours. Brain tissues were homogenized, lyophilized, and analyzed for 15N isotope ratio using elemental analysis coupled with stable isotope ratio mass spectrometry (IRMS).

### 4.6. The APP/PS1 Mouse Model

Five-month-old male C57BL/6 wild-type (WT) and APP/PS1 transgenic mice were purchased from Shanghai Nanmo Biological Co. (Shanghai, China). Mice were housed under a 12 h light/dark cycle with ad libitum access to water and food. All experiments were approved by the Animal Protection and Use Committee of Fudan University (China). All experiments were conducted in accordance with the guidelines of the Chinese Association for Laboratory Animal Sciences.

### 4.7. Grouping and Treatment Protocol for Mouse Models

Grouping and treatment of mouse models: Five-month-old male C57BL/6 (wild type, WT) mice were used as the control group (WT) (*n* = 6). AD mice were randomly divided into a model group (AD) (*n* = 6), positive drug group I (ST707) (*n* = 6), positive drug group II (gh625-Zn_7_MT3) (GM) (*n* = 6), and liposome group (AD808) (*n* = 6). After 6 weeks of intraperitoneal administration, the control group (WT) was injected with normal saline (200 μL/kg), the model group (AD) was injected with normal saline (200 μL/kg), the positive drug group I (ST707) was injected with 15 mg/kg, and the positive drug group II (GM) was injected with 2.5 mg/kg. Liposome group (AD808) was injected with AD808 200 μL/kg (equivalent to 15 mg/kg ST707 and 2.5 mg/kg gh625-Zn_7_MT3).

### 4.8. Assessment of Cognitive and Memory Functions in Mice

After 6 weeks of AD808 treatment, mouse learning and memory were assessed using the Mirrors water maze test. The apparatus consisted of a circular black-walled pool (diameter: 120 cm; height: 60 cm) divided into four equal quadrants, filled to 30 cm depth with water maintained at 25 °C. A 10 cm diameter escape platform was positioned in the target quadrant center, submerged 1 cm below the water surface. During testing, mice utilized spatial cues to locate the platform. Each trial began by placing mice in the water facing the wall at one of four starting quadrants. Mice underwent four daily trials for six consecutive days, with 15 min inter-trial intervals. The maximum trial duration was 90 s. Mice failing to find the platform within 90 s were gently guided to it and allowed to remain for 10 s. Daily escape latencies were averaged for each mouse. Following 6 training days, a probe test was conducted with the platform removed, starting mice from the quadrant farthest from the original platform location. Escape latency and platform crossings were video recorded over 120 s to assess spatial memory [22]. Water maze equipment was provided by Chengdu TME Technology Co., Ltd. (Chengdu, China).

### 4.9. Hematoxylin and Eosin (H&E) Staining

Brain tissues were fixed in 4% paraformaldehyde for 24 h, dehydrated through graded ethanol, and embedded in paraffin. 5 μm sections were deparaffinized with xylene, stained with hematoxylin (5 min) and eosin (2 min), then examined under a light microscope (Leica, Germany).

### 4.10. Cresyl Violet Staining for Nissl Granules

After standard fixation and paraffin embedding, sections were deparaffinized and stained with 0.1% cresyl violet (Nissl) solution for 10 min. Images were acquired using a light microscope (Leica, Germany).

### 4.11. TUNEL Assay of Neuron Apoptosis

Apoptotic cells were detected using the In Situ Cell Death Detection Kit (Roche). Deparaffinized sections were treated with proteinase K (20 μg/mL, 37 °C, 20 min), then incubated with TUNEL reaction mixture (1 h, 37 °C). After PBS washes, slides were mounted with antifade medium and imaged by confocal microscopy (Leica, Germany). Fluorescence intensity was quantified using ImageJ (1.8.0 NIH).

### 4.12. Aβ Plaque Histology

Sections were incubated with 0.3% (*w*/*v*) thioflavin-S in 50% ethanol (10 min, RT), differentiated in 50% ethanol (3 × 2 min), then covered with the coverslip for confocal microscopy. Plaque burden was calculated as thioflavin-S+ area/total area using ImageJ.

### 4.13. Biochemical Detection of Inflammatory Factors, Neurotrophic Factors, and Marker Aβ in AD Mouse Tissue

After the treatment of AD mice, whole blood was collected from the orbital veins and left at room temperature for 30 min before centrifugation. The supernatant was collected and immediately tested for AD marker Aβ. Whole brain tissue was collected from euthanized mice and mechanically homogenized to prepare a 10% homogenate. After centrifugation, the supernatant was collected for inflammatory factors TNF-α, IL-1β, IL-4, and IL-10, as well as neurotrophic factors BDNF, GDNF, and NGF-1.

### 4.14. Identification and Characterization of Microglial Subtypes in AD Mouse Tissue

Microglia, as key immune cells in the CNS, exhibit polarization states that significantly influence Alzheimer’s disease progression. Therefore qRT-PCR was employed to quantify expression changes of microglial polarization markers following ST707 treatment.

GAPDH: Forward: 5′-CCAGCCCAGCAAGGATACTG-3′

Reverse: 5′-GGTATTCGAGAGAAGGGAGGGC-3′

MCP-1: Forward: 5′-ACGCTTCTGGGCCTGTTGTT-3′

Reverse: 5′-CCTGCTGCTGGTGATTCTCT-3′

CD32: Forward: 5′-TCTTCCTAAAGTATCCCCTGGA-3′

Reverse: 5′-AAAGGGAGCTCCTTAACATGC-3′

Arg-1: Forward: 5′-CCAGCCCAGCAAGGATACTG-3′

Reverse: 5′-GGTATTCGAGAGAAGGGAGGGC-3′

TGF-β: Forward: 5′-CACCTGCAAGACCATCGACA-3′

Reverse: 5′-CATAGTAGTCCGCTTCGGGC-3′

### 4.15. Immunofluorescence Analysis of the MT3, TREM2, STING/IRF3, and PI3K/AKT Pathways in Brain Tissue

The STING/IRF3 and PI3K/AKT pathways in the brain tissue of AD mice was examined by immunofluorescence. Mouse brain tissue slices were deparaffinized and hydrated with xylene, antigen retrieval was performed, and the antigens were blocked with 5% fetal bovine serum (FBS) albumin for 2 h. Primary rabbit antibodies (1:50, Beijing Bio-Sense Biotechnology Co., Ltd., Beijing, China) were added and incubated overnight at 4 °C. The next day, after rewarming at 37 °C, the secondary antibody (1:100) was added dropwise, incubated at 37 °C for 2 h, and mounted with an anti-fade fluorescent mounting medium containing DAPI (protected from light). Images were captured using a fluorescence microscope (BX51, Olympus, Japan), and the average fluorescence intensity was quantified using ImageJ software.

### 4.16. Data Analysis

Experimental data were plotted using GraphPad Prism 9.5 and expressed as mean ± standard deviation (mean ± SD). The Q-Q plot analysis was conducted to confirm that our data satisfied the normality assumption. One-way and two-way ANOVA was conducted using SPSS (version 19.0), followed by Tukey’s test with significance levels denoted as * *p* < 0.05 and ** *p* < 0.01.

## 5. Conclusions

In this study, the APP/PS1 double transgenic AD mouse model was used to investigate the pharmacodynamic effects and mechanisms of the dual-target liposome complex AD808 in Alzheimer’s disease. AD808 effectively prevented AD progression in mice, improved learning and memory abilities, reduced neuronal apoptosis in the hippocampus, and alleviated neuropathological damage. Its efficacy was significantly superior to that of the innate immunomodulator ST707 or the metal homeostasis regulator MT3 alone.

Based on these findings, it proposed the pharmacological mechanism underlying its anti-AD effects. The gh625-Zn_7_MT3 modification on the surface of the AD808 liposome complex regulates metal homeostasis by chelating overloaded Cu^2+^ ions in the brain, reducing chronic toxicity from Cu accumulation, and inhibiting Aβ synthesis. On one hand, AD808 modulates TREM2 overexpression in microglia and reduces Aβ plaque deposition in the brain, thereby exerting neuroprotective effects. On the other hand, AD808 activates the STING/IRF3 and IRF3/PI3K/AKT pathways, regulating microglial phenotypic transformation. This promotes M1-to-M2 polarization, releasing anti-inflammatory factors to mitigate chronic inflammation and neurotrophic factors to repair damaged neurons, thereby reducing ischemia-induced neuronal injury and enhancing brain tissue recovery.

These results demonstrate that, compared to ST707 and gh625-Zn_7_MT3, the AD808 liposome complex exhibits superior efficacy in improving learning and memory abilities in AD mice and ameliorating neuronal apoptosis and hippocampal tissue damage. This study provides innovative insights and a theoretical foundation for developing novel anti-AD drugs.

## Figures and Tables

**Table 1:** Characterization of liposomal complex AD808.

Type	Size (nm)	Zeta (mv)	PDI	EE (%)	DL (%)	Protein Content (μg/mL)
Lip-blank	182.6 ± 0.6	−0.43 ± 1.2	0.114			
AD808	198.1 ± 1.2	−8.1 ± 4.9	0.119	88.7 ± 5.3	8.8 ± 0.1	231.4 ± 16.5

## Data Availability

The original data are available from the corresponding authors upon reasonable request.

## Abbreviations

The following abbreviations are used in this manuscript:

HSPC	Hydrogenated soybean phosphatidylcholine
CHO	Cholesterol
DSPE-PEG2000	Phospholipid-methoxy polyethylene glycol
DSPE-PEG2000-Mal	Maleimide-phospholipid-methoxy polyethylene glycol
Aβ	β-amyloid
TGF-β	Transforming growth factor-β
MCP-1	Monocyte chemoattractant protein-1
